# Postoperative outcomes of transperitoneal versus retroperitoneal robotic partial nephrectomy: a propensity-score matched comparison focused on patient mobilization, return to bowel function, and pain

**DOI:** 10.1007/s11701-024-01860-7

**Published:** 2024-02-28

**Authors:** Riccardo Bertolo, Francesco Ditonno, Alessandro Veccia, Vincenzo De Marco, Filippo Migliorini, Antonio Benito Porcaro, Riccardo Rizzetto, Maria Angela Cerruto, Riccardo Autorino, Alessandro Antonelli, PubMed-indexed collaborators, Damiano D’Aietti, Sebastian Gallina, Davide Brusa, Michele Boldini, Sonia Costantino, Alberto Baielli, Francesca Montanaro, Francesco Artoni

**Affiliations:** 1 Department of Urology, University of Verona, Azienda Ospedaliera Universitaria Integrata, AUOI Verona, Borgo Trento Hospital, Piazzale Aristide Stefani 1, 37126 Verona, Italy; 2 Department of Urology, Rush University Medical Center, Chicago, IL USA

**Keywords:** Nephrectomy, Renal neoplasm, Robotic, Approach, Retroperitoneal

## Abstract

Literature meta-analyses comparing transperitoneal versus retroperitoneal approach to robotic partial nephrectomy (RPN) suggested some advantages favoring retroperitoneoscopy. Unfortunately, patient-centered data about mobilization, canalization, pain, and use of painkillers remained anecdotally reported. The present analysis aimed to compare transperitoneal versus retroperitoneal RPN focusing on such outcomes. Study data including baseline variables, perioperative, and postoperative outcomes of interest were retrieved from prospectively maintained institutional database (Jan 2018–May 2023) and compared between treatment groups (transperitoneal versus retroperitoneal). Propensity score matching was performed using the STATA command *psmatch2* considering age, sex, body mass index, previous abdominal surgery, RENAL score, tumor size and location, and cT stage. The logit of propensity score was used for matching, with a 1:1 nearest neighbor algorithm, without replacement (caliper of 0.001). A total of 442 patients were included in the unmatched analysis: 330 underwent transperitoneal RPN 112 retroperitoneal RPN. After propensity score, 98 patients who underwent retroperitoneal RPN were matched with 98 patients who underwent transperitoneal RPN. Matched cohorts had comparable patients’ demographics and tumor features. We found similarity between the two laparoscopic accesses in all outcomes but in blood loss, which favored retroperitoneoscopic RPN (median 150 (IQR 100–300) versus 100 (IQR 0–100) ml, *p* = 0.03). No differences were found in terms of time to mobilization with ambulation, return to complete bowel function, postoperative pain, but higher painkillers consumption was reported after transperitoneal RPN (*p* < 0.004). The present study compared the transperitoneal versus the retroperitoneal approach to RPN, confirming the similarity between the two approaches in all perioperative outcomes. Based on our findings, the choice of the surgical approach to RPN may remain something that the surgeon decides.

**Supplementary Information:**

The online version contains supplementary material available at 10.1007/s11701-024-01860-7.

## Introduction

Robotic partial nephrectomy (RPN) has become an established surgical management of small renal masses [1]. It offers minimally invasive access as laparoscopy, but a much easier learning curve [2, 3]. RPN can be carried out via a transperitoneal or retroperitoneal approach. The choice between the two laparoscopic approaches is usually based on surgeon preference.

The transperitoneal approach has known broader diffusion due to the larger working space allowed and the supposed better recognition of anatomical structures.

On the other hand, the retroperitoneal approach would have the ideal advantage of avoiding the peritoneal cavity and, thus, the potential adhesions from previous transabdominal surgeries. Hypothetically, it gives easier access to posterior tumors. Moreover, the retroperitoneal approach grants direct access to the hilar structures without any kidney mobilization. Nevertheless, the confined space and the less familiar landmarks have limited the popularity of retroperitoneal approach across the urological community [4].

The meta-analyses of literature published on the topic all go in the same direction, suggesting that the retroperitoneal approach may provide advantages in terms of shorter operative time, lower blood loss, and less perioperative morbidity [4–6].

Unfortunately, most of the available studies lack patient-centered data about mobilization, canalization, postoperative pain, and the use of painkillers [4].

The present analysis aimed to explore and compare transperitoneal and retroperitoneal approaches to RPN, focusing on such outcomes.

## Methods

### Patient selection

This retrospective comparative study was conducted to evaluate the impact of the laparoscopic access used to perform RPN (transperitoneal versus retroperitoneal) on postoperative patient mobilization, the return to bowel function, the pain at discharge, and the management of the eventual pain. All procedures were conducted in accordance with ethical standards and the principles of the Declaration of Helsinki.

The study data were retrieved from the prospectively maintained institutional database relative to patients undergoing kidney surgery for renal masses. The study included all consecutive patients who underwent RPN for renal masses with either a transperitoneal or a retroperitoneal approach between January 2018 and May 2023 at our Institution.

Exclusion criteria encompassed patients with a history of prior partial nephrectomy, patients with multiple ipsilateral tumors, and patients with tumor(s) diagnosed in solitary kidney.

In addition, patients with missing data regarding the outcomes of interest and/or incomplete follow-up data were excluded.

### Surgical technique

All surgeries were performed by experienced robotic surgeons (number of robotic surgeries completed before the study started > 150) using the da Vinci Surgical System [7].

A standardized surgical approach, including the hilar dissection, the preparation of the renal lesion, and the use of intraoperative ultrasonography, was maintained across all cases, regardless of the laparoscopic approach. Of interest for the purpose of the present study, the decision to perform RPN with a transperitoneal versus a retroperitoneal approach was based on the surgeon’s preference relative to the lesion location. Retroperitoneoscopic RPN was performed as previously described by our group [8].

### Outcomes measurements

Demographic, clinical, intraoperative, and postoperative data were prospectively collected on the institutional electronic medical records. Data retrieved included (1) baseline patients’ demographics, body mass index (BMI), comorbidities as classified according to the Charlson’s comorbidity index (CCI) [9], ability to perform activities of daily living (as classified according to the Barthel index) [10], history of previous abdominal surgery, preoperative hemoglobin (Hb), serum creatinine (SCr), estimated glomerular filtration rate (eGFR) (calculated by using the Chronic Kidney Disease Epidemiology Collaboration formula) [11], clinical tumor size, location, and RENAL nephrometry score [12]; (2) intraoperative variables such as estimated blood loss (EBL), operative time, management of the renal hilum (off-clamp versus on-clamp), warm ischemia time duration (WIT), eventual bolstering and/or use of hemostatic agents, and occurrence of intraoperative complications; and (3) postoperative outcomes included eventual complications, graded according to the Clavien–Dindo classification (complications with Clavien–Dindo grade ≥ III were considered as “major complications”) [13], length of stay, 90 day readmission rate, reassessment of SCr and eGFR on postoperative day one (1st POD), at discharge, and at the last follow-up. Specifically, for the purpose of the study, data regarding mobilization with ambulation, return to bowel function (defined as the time to complete canalization), evaluation of perceived pain at discharge (assessed by the visual analogue scale—VAS), and the pain management strategy (grams of paracetamol prescribed during hospitalization, and/or eventual need for additional non-steroidal anti-inflammatory drugs—NSAIDs—and/or use of opioids for analgesia) were retrieved.

### Statistical analysis

Statistical analysis was conducted according to published guidelines [14, 15]. The patients were stratified into two groups according to the laparoscopic approach used to perform RPN (transperitoneal versus retroperitoneal). The medians and the interquartile ranges (IQR) were used to summarize continuous variables. Frequencies and proportions were used to report categorical variables. Before matching, descriptive analyses comparing the two groups were conducted using the Mann–Whitney *U* test and the Fisher’s exact test, as appropriate. Propensity score matching was performed using the STATA command *psmatch2*, to control for potential differences at baseline between patients in the transperitoneal and the retroperitoneal cohorts. Logistic regression with the outcome of the surgical approach was used to calculate the propensity scores. Patients were matched for age, sex, BMI, history of previous abdominal surgery, RENAL score, tumor size and location (polar versus mediorenal, hilar versus non-hilar, and posterior versus non-posterior location), and cT stage. The logit of the propensity score was used for matching, with a 1:1 nearest neighbor algorithm, without replacement, with a caliper of 0.001. After propensity score matching, descriptive statistics were conducted for the matched cohorts using the same methods employed to analyze the unmatched cohorts. The analysis of surgical and postoperative outcomes was performed using the same approach. The Stata^®^ 17.0 software (StataCorp LLC, College Station, TX, USA) was used for statistical analysis with statistical significance set at *p* value < 0.05.

## Results

### Study population

Overall, a total of 442 patients met the inclusion criteria and were included in the unmatched analysis. Of these, 330 underwent RPN with a transperitoneal approach and 112 with a retroperitoneal approach. No differences were observed when comparing the unmatched groups at baseline but in the proportion of patients who had undergone previous abdominal surgery (11 versus 26%, transperitoneal versus retroperitoneal, respectively, *p* < 0.001), and the proportion of patients who had anteriorly located tumors (49 versus 28%, transperitoneal versus retroperitoneal, respectively, *p* = 0.02) (Supplementary Table 1). After the propensity score, 98 patients who underwent retroperitoneal RPN were matched with 98 patients who underwent transperitoneal RPN. The matched cohorts had comparable patients’ and clinical tumor features (Table 1).

**Table 1:** Baseline features after propensity score matching

	Transperitoneal (98)	Retroperitoneal (98)	*p* value
Age, years			
Median (IQR)	69 (62–73)	64 (53–72)	0.3
Sex			
No. males (%)	55/98 (56%)	63/98 (64%)	0.5
BMI			
Median (IQR)	27.1 (24.2–29.3)	25.5 (24.5–30)	0.1
CCI			
Median (IQR)	2 (1–3)	2 (2–4)	0.1
Barthel index			
Median (IQR)	100 (100–100)	100 (100–100)	0.9
Previous abdominal surgery			
No. (%)	16/98 (16%)	12/98 (12%)	0.5
Serum creatinine			
Median (IQR)	0.98 (0.81–1.22)	0.87 (0.79–1.13)	0.1
eGFR			
Median (IQR)	82 (60–91)	78 (65–93.3)	0.6
Clinical tumor size, cm			
Median (IQR)	3.1 (2.3–3.6)	3 (2–3.5)	0.8
RENAL nephrometry score			
Median (IQR)	6 (5–8)	7 (6–7)	0.3
Location of renal mass			
No. hilar (%)	12/98 (12%)	8/98 (8%)	0.3
No. anterior (%)	31/98 (32%)	34/98 (35%)	0.7
No. polar (%)	33/98 (33%)	34/98 (35%)	1

Continuous variables were compared using Mann–Whitney *U* test

Categorical variables were compared using Fisher’s exact test

*BMI* Body mass index, *CCI* Charlson Comorbidity Index, *eGFR* estimated glomerular filtration rate

### Surgical outcomes

Treatment groups were not statistically different in terms of operative time (*p* = 0.1), WIT (*p* = 0.3), proportions of patients managed with on- versus off-clamp technique (*p* = 0.5), use of bolster (*p* = 0.5) and of hemostatic agents (*p* = 0.6). Conversely, retroperitoneal RPN had significantly lower EBL (*p* = 0.03) (Table 2).

**Table 2:** Surgical outcomes

	Transperitoneal (98)	Retroperitoneal (98)	*p* value
On-clamp, no. (%)			
Yes	44/98 (50%)	51/98 (52%)	0.5
Ischemia time, min			
Median (IQR)	18 (5–21)	14 (8–17)	0.3
Estimated blood loss, mL			
Median (IQR)	150 (100–300)	100 (0–100)	**0.03**
Operative time, min			
Median (IQR)	154 (107–184)	128 (93–166)	0.1
Intraoperative complication, no. (%)			
Yes	1/98 (1%)	0/98 (0%)	0.5
Placement of drainage, no. (%)			
Yes	55/98 (86%)	67/98 (68%)	0.5
Bolstering, no. (%)			
Yes	63/98 (65%)	68/98 (69%)	0.5
Use of hemostatic agent, no. (%)			
Yes	90/98 (92%)	82/98 (84%)	0.6

Bold p-values < 0.05, indicating the statistical significance of the finding

Continuous variables were compared using Mann–Whitney *U* test

Categorical variables were compared using Fisher’s exact test

### Postoperative outcomes

Comparable rates of overall postoperative complications were observed between the groups (21% versus 31%, transperitoneal versus retroperitoneal, *p* = 0.09). Major complications were 3% and 4%, respectively, with no differences (*p* = 0.7). Median length of stay was not statistically different (*p* = 0.9).

No differences between the two approaches were observed in terms of number of patients achieving mobilization with ambulation (*p* = 0.6), and complete canalization after surgery (*p* = 0.7). As concerning pain, a similar median (IQR) VAS was observed (*p* = 0.7). Three percent of the patients in the transperitoneal group versus 8% of patients in the retroperitoneal group had a pain score ≥ 2 according to VAS at discharge, with no statistically significant difference (*p* = 0.2). Groups were non-statistically different in terms of grams of paracetamol administered during hospitalization (*p* = 0.7), but the need for additional NSAIDs (*p* = 0.002) and opioids (*p* = 0.004) was statistically significantly higher for patients who underwent transperitoneal surgery (Table 3).

**Table 3:** Postoperative outcomes

	Transperitoneal (98)	Retroperitoneal (98)	*p* value
Postoperative complications, no. (%)			
Overall	21/98 (21%)	30/98 (31%)	0.1
Clavien–Dindo grade ≥ 3	6/207 (3%)	4/108 (4%)	0.7
Transfusion	4/98 (4%)	0/98 (0%)	0.5
Embolization	0/98 (0.5%)	0/98 (0%)	–
Management of complications, no. (%)			
Transfusion	4/98 (4%)	0/98 (0%)	0.5
Embolization	0/98 (0.5%)	0/98 (0%)	–
Blood tests on 1st POD, median (IQR)			
Hemoglobin	13.2 (11.5–13.8)	13.2 (12–13.8)	0.7
Serum creatinine	1.05 (0.96–1.54)	0.99 (0.84–1.16)	.06
eGFR	65.5 (46–83)	66.35 (45.8–82.5)	0.5
Blood tests at discharge, median (IQR)			
Hemoglobin	12.4 (11.2–13.9)	13 (11.9–13.7)	0.5
Serum creatinine	1.02 (0.87–1.43)	0.92 (0.78–1.08)	0.1
eGFR	73 (52–98)	72 (54–91)	0.6
Blood tests at last follow-up*, median (IQR)			
Serum creatinine	1.01 (0.82–1.46)	0.91 (0.78–0.99)	0.1
eGFR	69 (52–87)	66 (52–84)	0.6
Mobilization w/deambulation on POD			
Median (IQR)	1 (1–2)	1 (1–2)	0.2
> POD day 2, no. (%)	4/98 (4%)	0/98 (0%)	0.6
Canalization on POD			
Median (IQR)	3 (2–4)	3 (2–4)	0.8
> POD day 2, no. (%)	7/98 (7%)	3/98 (3%)	0.7
Pain (VAS) at discharge,			
Median (IQR)	1 (0–3)	2 (1–3)	0.7
Pain management			
Paracetamol, grams, median (IQR)	5.5 (4–6.5)	5 (3–8)	0.7
Additional NSAIDs, *n* (%)	26/98 (26%)	9/98 (9%)	**0.002**
Additional opioids, *n* (%)	10/98 (11%)	0/98 (0%)	**0.004**
Duration of hospitalization, days			
Median (IQR)	4 (4–5)	5 (4–5)	0.9
90 days readmission			
Yes, *n* (%)	0/98 (0%)	0/98 (0%)	–

Bold p-values < 0.05, indicating the statistical significance of the finding

Continuous variables were compared using Mann–Whitney *U* test

Categorical variables were compared using Fisher’s exact test

*POD* postoperative day, *eGFR* estimated Glomerular Filtration Rate, *LOS* length of stay, *VAS* visual analogue scale

*Median follow-up: 14 months (12–22)

## Discussion

The present study compared the transperitoneal versus the retroperitoneal approach to RPN, confirming a substantial similarity between the two laparoscopic routes except for blood loss, which favored retroperitoneoscopic RPN. The specific purpose of the study was to focus on patient-centered outcomes, which included postoperative mobilization with ambulation, return to complete bowel function, postoperative pain, and its management with painkillers. No differences were found in terms of these outcomes, except for painkiller consumption.

The simple comparison between transperitoneal and retroperitoneal laparoscopic surgical access to RPN is not inherently original. Consequently, numerous comparative studies have been published since the advent of robotic platforms, starting in 2013 [16, 17]. Within the last decade, several research groups have summarized the available evidence in the field by publishing literature meta-analyses. Almost all the published studies have consistently reported certain advantages in terms of perioperative outcomes when a retroperitoneal approach is adopted. These include faster surgery, minimized blood loss, and shortened duration of hospitalization [4–6].

The sole statistically significant difference identified in terms of perioperative outcomes in the context of the present study has been the lower blood loss observed in the retroperitoneoscopic cohort (median 150 (IQR 100–300) versus 100 (IQR 0–100) ml, transperitoneal versus retroperitoneal approach, respectively, *p* = 0.03), consistent with previous experiences. Some authors have attributed the difference in blood loss to the requirement for a lesser extent of tissue dissection during retroperitoneal access [18].

Nevertheless, the clinical impact of these differences appears to be negligible, as both techniques demonstrate comparable oncological efficacy and renal functional outcomes—the primary endpoints of partial nephrectomy.

Regrettably, the primary limitation of the existing literature lies in the predominance of either retrospective or prospective non-randomized designs in published studies. However, it’s worth noting that despite this limitation, some studies exhibit good quality by incorporating controls for potential confounders (i.e., a matched-paired analysis).

Furthermore, nearly all the published studies lack a prospectively detailed collection of what the authors consider more interesting outcome measurements. This is particularly notable when comparing interventions that involve the same surgical steps but are conducted through different laparoscopic accesses.

The most up-to-date and largest cumulative analysis of comparative studies about transperitoneal versus retroperitoneal RPN pointed out that “*the impact on quality of life indices remains to be determined”* [4]. What are we talking about? To the best of our knowledge, only a propensity score-matched analysis born within the RECORd 2 project (comparing > 400 patients per treatment group) reported data about bowel canalization and found a shorter time of return to bowel function with retroperitoneal access (median 3 (IQR 2–5) versus 2 (IQR 1–3) days, transperitoneal versus retroperitoneal, respectively, *p* < 0.0001 [19]. The finding is not unexpected. As such, during the retroperitoneal approach to nephron-sparing surgery, the bowel remains untouched, whereas the transperitoneal approach necessitates some degree of colonic dissection, carrying the potential for postoperative ileus.

Our database has the strength of a prospective data collection of specific postoperative outcomes which have remained anecdotally reported by previous literature about the topic, namely, beyond the just mentioned return to bowel function, the patient mobilization with ambulation after surgery, the perceived pain, and its management with painkillers.

The null hypothesis of our analysis posited that, due to its more targeted anatomical approach, the retroperitoneal method could minimize the impact of surgery, potentially resulting in lower pain, faster mobilization, and a quicker return to bowel function. However, our results did not support the null hypothesis, as the two approaches were comparable in these outcome measurements as well. It’s worth noting that our dataset included the Barthel index, collected preoperatively, indicating that all patients considered in our analysis had a comparable ability to perform activities of daily living before the surgery. Nevertheless, we observed a significantly higher adoption of painkillers for patients who underwent surgery via a transperitoneal approach. Although this finding contradicts the pain perceived, as assessed by the VAS in the present study, the increased consumption of painkillers could suggest the potential for lower invasiveness associated with the retroperitoneal approach. While the VAS is widely used and has been shown to be reliable and valid for pain assessment, no single tool is perfect for all situations. Pain is a complex and subjective experience, and different individuals may interpret and express their pain differently. In some cases, the combination of pain assessment tools should be preferred to gather a more comprehensive understanding of the pain experience.

We acknowledge the limitations of our study. First, although the prospective granular data collection, the study design was retrospective, which led to potential imbalances between the cohorts despite propensity score matching: this is not always the ideal approach for establishing causal inference, as it does not account for unobserved confounders. This may explain why we failed at finding any significant difference between the two approaches. Second, the study was conducted in the setting of a tertiary referral institution for nephron-sparing surgery. This could limit the generalizability of our findings. But, while most centers have limited/no expertise with the retroperitoneal route (we believe this could compromise a fair comparison between the two techniques), we pride ourselves on extensive experience with both the approaches. We feel this is another strength of the present analysis.

On the other hand, it is interesting to note how surgeons performing single-port RPN in the United States by using a novel purpose-built robot are more likely to choose a retroperitoneal approach. This is likely due to one of the key features of the “SP” robot, which is well suited to work within narrow spaces such as the retroperitoneum, facilitates access and docking, and reduces the need for dissecting several anatomical structures before performing the resection of the renal mass [20]. Thus, the advent of single-port robotic surgery in Europe could expand the interest towards retroperitoneal access for RPN [21].

Finally, to the best of our knowledge, this is the first study investigating some unconventional quality of life-related outcome measurements, relying upon a decent sample size relative to the topic investigated. We sincerely believe this is a plus of our effort which adds to previous literature.

## Conclusions

The present study compared the transperitoneal versus the retroperitoneal approach to RPN, confirming the similarity between the two routes in all perioperative outcomes but in blood loss and consumption of additional painkillers, which favored retroperitoneoscopic RPN. No differences were found in terms of postoperative patient mobilization with ambulation, return to complete bowel function, and postoperative pain.

## Supplementary Information

Below is the link to the electronic supplementary material.

Supplementary file1 (DOCX 24 KB)

## Data Availability

The data that support the findings of this study are available on request from the corresponding author, R.B
