# Supplementary material for: Postoperative outcomes of transperitoneal versus retroperitoneal robotic partial nephrectomy: a propensity-score matched comparison focused on patient mobilization, return to bowel function, and pain
Source: J Robot Surg. 2024 Feb 28;18(1):96. doi: 10.1007/s11701-024-01860-7 (PMC10899314; doi:10.1007/s11701-024-01860-7)
Supplement: Supplementary file 1 — Supplementary file1 (DOCX 24 KB) [file 11701_2024_1860_MOESM1_ESM.docx]

|  | Transperitoneal  (330) | Retroperitoneal  (112) | p-value |
| --- | --- | --- | --- |
| Age, years  Median (IQR) | 63 (55-72) | 62.5 (55-72) | 0.9 |
| Sex  No. Male (%) | 234/330 (71%) | 75/112 (67%) | 0.3 |
| BMI  Median (IQR) | 26.5 (23.8-29) | 25.9 (24.5-30) | 0.4 |
| CCI  Median (IQR) | 2 (2-3) | 2 (2-3) | 0.9 |
| Barthel index  Median (IQR) | 100 (100-100) | 100 (100-100) | 0.9 |
| Previous abdominal surgery  No. (%) | 36/330 (11%) | 29/112 (26%) | **<.001** |
| Serum creatinine  Median (IQR) | 0.91 (0.79-1.07) | 0.87 (0.79-1.17) | 0.8 |
| eGFR, ml/min  Median (IQR) | 86 (72-96) | 84 (70-96) | 0.7 |
| Clinical Tumor Size, cm  Median (IQR) | 3 (2.2-4.6) | 2.8 (2-3.5) | 0.1 |
| RENAL Nephrometry score  Median (IQR) | 7 (5-9) | 7 (6-8) | 0.9 |
| Location of renal mass  No. Hilar (%)  No. Anterior (%)  No. Polar (%) | 48/330 (15%)  161/330 (49%)  172/330 (52%) | 10/112 (9%)  43/112 (38%)  66/112 (59%) | .1  **.02**  .2 |

**Table 1.** Baseline features before propensity score matching

Abbreviations: BMI (Body mass index); CCI (Charlson Comorbidity Index); eGFR (estimated glomerular filtration rate).

Continuous variables were compared using Mann-Whitney U-test

Categorical variables were compared using Fisher’s exact test
